# Detection and Genetic Characterization of Red-Spotted Grouper Nervous Necrosis Virus and a Novel Genotype of Nervous Necrosis Virus in Black Sea Bass from the U.S. Atlantic Coast

**DOI:** 10.3390/v17091234

**Published:** 2025-09-10

**Authors:** Jan Lovy, Miriam Abbadi, Anna Toffan, Nilanjana Das, James N. Neugebauer, William N. Batts, Peter J. Clarke

**Affiliations:** 1 U.S. Geological Survey, Western Fisheries Research Center, 6505 NE 65th Street, Seattle, WA 98115, USA; bbatts@usgs.gov; 2 Istituto Zooprofilattico Sperimentale delle Venezie, Viale dell’Università 10, 35020 Legnaro, PD, Italy; mabbadi@izsvenezie.it (M.A.); atoffan@izsvenezie.it (A.T.); 3 Department of Microbiology, Oregon State University, Corvallis, OR 97331, USA; dasni@oregonstate.edu; 4 Office of Fish and Wildlife Health and Forensics, New Jersey Fish and Wildlife, Oxford, NJ 07863, USA; 5 Bureau of Marine Fisheries, New Jersey Fish and Wildlife, Nacote Creek, NJ 08205, USA; peter.clarke@dep.nj.gov

**Keywords:** nervous necrosis virus, viral encephalopathy and retinopathy, viral nervous necrosis, genetics, black sea bass, United States, Atlantic

## Abstract

Nervous necrosis virus (NNV) causes a neurologic disease in a wide range of marine fish and poses serious disease risks to marine aquaculture worldwide. Little is known about the presence of NNV along the Atlantic coast of the United States, aside from the presence of barfin flounder nervous necrosis virus (BFNNV) in coldwater species in the northern part of this range. Herein we conducted surveillance for NNV from 2020 to 2022 in the mid-Atlantic region of the United States in black sea bass *Centropristis striata*, a serranid fish that is found throughout the eastern U.S. coast. Molecular detection methods have identified and characterized red-spotted grouper nervous necrosis virus (RGNNV) sequences at low prevalence throughout the years. Further, in 2022, a higher prevalence of a novel NNV genotype, tentatively named black sea bass nervous necrosis virus (BSBNNV), was characterized for the first time. Though virus isolation was unsuccessful, this study was the first to genetically identify NNV in this region and in this species. These findings highlight the need for further research on NNV to understand epidemiology and virulence in the context of marine fisheries and an emerging marine aquaculture industry in the United States.

## 1. Introduction

Viral encephalopathy and retinopathy, also known as viral nervous necrosis (VNN), is caused by nervous necrosis virus (NNV), a neurotropic RNA virus belonging to the genus *Betanodavirus* within the family Nodaviridae [1,2]. The disease VNN causes clinical signs in fish, including whirling, hyperactivity, and/or lethargy, which is associated with vacuolating lesions within the brain and retina [3]. NNV has a relatively small genome (total of 4.5 kb) comprising two single-stranded, positive-sense molecules, including the RNA1 (3.1 kb) encoding the RNA-dependent RNA polymerase and the RNA2 (1.4 kb) encoding the viral capsid [4,5,6]. NNV has a wide host range in marine fish species with four unique genotypes recognized, based on genetic sequences within a highly variable region (T4) of the RNA2 gene [7]. These include red-spotted grouper nervous necrosis virus (RGNNV), striped jack nervous necrosis virus (SJNNV), tiger puffer nervous necrosis virus (TPNNV), and barfin flounder nervous necrosis virus (BFNNV) [7]. These genotypes are associated with certain host species [8] and optimal replication temperatures [9,10,11]; as such, they may be confined to certain geographic ranges. For example, BFNNV occurs in cooler regions including Japan, northern Europe, and northern North America [12,13,14,15], whereas RGNNV occurs in tropical and temperate fishes across a wider range, as reviewed by [16]. Though these four genotypes have been well described and accepted as viral species by the ICTV [1], a high level of genetic diversity and additional genotypes are still being recognized. This is evidenced by other recently described genotypes that are awaiting formal recognition as viral species [17,18].

**Figure 1:**
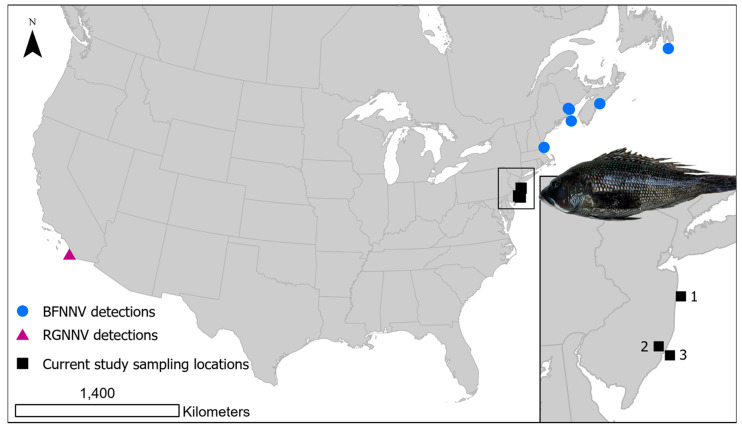
Sites of black sea bass *Centropristis striata* collection from coastal New Jersey for this study, including 1. Sea Girt Reef; 2. Rutgers Marine Field Station; 3. Little Egg Reef. Previous reports of nervous necrosis virus detected in North America are indicated; barfin flounder nervous necrosis virus (BFNNV) [13,14,26]; red-spotted grouper nervous necrosis virus (RGNNV) [27].

Viral nervous necrosis is considered one of the most serious disease threats to global marine aquaculture. For example, in all regions of the Mediterranean Sea, VNN is viewed as the most important contributor to disease in the farming of European sea bass *Dicentrarchus labrax* and gilthead sea bream *Sparus aurata* [19,20,21]. Serious disease in marine aquaculture has also been reported in Asia, including Japan [15,22,23] and Taiwan [24], and in Norwegian Atlantic halibut *Hippoglossus hippoglossus* juvenile rearing facilities [25]. In North America, BFNNV caused disease issues in farmed Atlantic cod *Gadus morhua* and haddock *Melanogrammus aeglefinus* from Atlantic Canada and the northeastern United States [14,26], while in the western United States, RGNNV was associated with an outbreak in farmed white sea bass *Atractoscion nobilis* from the Pacific coast in California [27] (Figure 1). Little is known of NNV prevalence outside these specific regions in the U.S. Considering the high priority to expand marine offshore aquaculture in the U.S. to offset the capture of wild fisheries [28], understanding risks from NNV in the region may help develop fish health planning as the industry is established. Because of limited biosecurity control in the offshore environment, viruses and other pathogens may be introduced to marine aquaculture from wild fish reservoirs [29], emphasizing the need to understand pathogen distribution and prevalence in wild fish.

Much of the attention to NNV has been within aquaculture settings, while the impacts of this virus on wild fish are less known. Wild fish are considered natural reservoirs for NNV, with BFNNV detected in wild winter flounder *Pleuronectes americanus*, in Atlantic Canada [13] and RGNNV in wild groupers *Epinephelus* spp. and European sea bass in regions of marine aquaculture in the Mediterranean Sea [30,31]. Virulence of the virus is known to be highest in larval and juvenile stages [32,33], though mortality in these early stages of fish may be easily missed in the wild. Disease in wild adult fish has been associated with erratic swimming, lethargy, skin and fin erosions, eye lesions, and hyperinflated swim bladders in adult groupers *Epinephelus* spp. and European sea bass [30,31]. In the present study, we investigated NNV in wild black sea bass *Centropristis striata*, an important species that is fished commercially and recreationally in the mid-Atlantic U.S. In addition to supporting fisheries, black sea bass are considered an ideal commercially ready species for U.S. marine aquaculture [34].

Black sea bass, within the family Serranidae (sea basses and groupers), are demersal fishes with a preference for warm temperate waters with a range from Maine to Florida in the northwestern Atlantic Ocean [35]. The species is managed in two distinct populations, including a mid-Atlantic population that spans from the Gulf of Maine to Cape Hatteras, North Carolina, and a southern population that spans from Cape Hatteras to Florida [36,37]. The mid-Atlantic population feed and spawn in coastal areas between May and October and in the fall migrate offshore toward the continental shelf and south to the Chesapeake Bight to overwinter in warmer waters [38,39]. With a preference for structured habitat, and the middle Atlantic Bight comprising a relatively flat topology, black sea bass have benefited from man-made reefs in this region, such as shipwrecks and artificial reefs [40]. The success in artificial reefs supporting higher fish abundance and species diversity may also influence transmission factors for certain infectious agents. For example, *Lernaeenicus radiatus*, a pennellid copepod with a complex life cycle, was found at a higher prevalence and infection intensity in black sea bass from artificial reef sites compared to non-structured habitats. This is likely a result of increased transmission factors related to increased fish density and biodiversity favored in reef environments [41].

In the present investigation, surveillance for NNV has been conducted alongside an artificial reef black sea bass monitoring survey led by the Bureau of Marine Fisheries, New Jersey Fish and Wildlife. Between 2020 and 2022, 865 adult black sea bass were screened with molecular methods for NNV, documenting the virus in this species for the first time. Genetic sequence analysis conducted herein documents the presence of RGNNV, not previously detected on the Atlantic coast of North America, and a previously undescribed NNV genotype, indicating the role of NNV as a potential pathogen within the region that is highly relevant to fisheries and emerging aquaculture.

## 2. Materials and Methods

### 2.1. Fish Collection and Environmental Data

Across 2020–2022, adult black sea bass were collected in collaboration with the artificial reef monitoring trap survey conducted by the Bureau of Marine Fisheries, New Jersey Fish and Wildlife. Artificial reefs are made of concrete, dredge rock, decommissioned barges, old ships, tanks, subway cars, and steel demolition debris to provide structured habitat for demersal fishes. Fish were captured from two artificial reefs, Sea Girt Reef and Little Egg Reef, both located off the shore of New Jersey (Figure 1). Little Egg Reef is 3.2 km^2^ in size and located 6.1 nautical km offshore with a depth of 15–18 m. Sea Girt Reef is 2.25 km^2^ in size, located 5.6 nautical km offshore, and has a depth range of 17–22 m.

For fish collection, 22 un-baited ventless traps measuring 112 L × 58 W × 38 H (cm) were randomly placed throughout the reef sites between April and November. The traps were checked within 7 d of deployment. After capture, fish were placed on ice and transferred to the Pequest Aquatic Animal Health Laboratory, Oxford, New Jersey for tissue sampling within 24 h of capture. Acceptable methods for fish handling/sampling were under the direction of the New Jersey Fish and Wildlife and the Federal Sportfish and Restoration Project (FW-69-R21). Total length, weight, and sex was recorded for each fish. In 2020, in addition to NNV screening, fish were additionally screened for other general systemic viruses. For this general screening, spleen and kidney were aseptically collected from 146 fish with up to five fish pooled per sample to screen for viruses by isolation using viral cell culture assays, as further described below. Between 2020 and 2022, 865 adult black sea bass were screened for NNV using a two-step reverse transcription real-time PCR (rRT-PCR), further described below. Brain was aseptically collected into a 2 mL microcentrifuge tube. Instruments were disinfected with bleach, water, ethanol, and flame between fish samples. Tissue samples were immediately frozen at −80 °C. The remaining brain and eye tissue was collected into a sterile sample bag and kept frozen at −80 °C.

In 2020 only, young-of-the-year (YOY) black sea bass were also collected between March 24 and August 4 in collaboration with the Rutgers University Marine Field Station. Un-baited Gee wire mesh traps were deployed at the marine field station boat basin and sampled twice weekly, as previously described [42]. A total of 116 YOY black sea bass were collected, euthanized with an overdose (200 mg/L) of buffered MS-222 (Syndel, Ferndale, WA, USA), and frozen at −20 °C until further processing. Fish were sampled while frozen by excising and pooling brain tissue from 5 fish into 2 mL microcentrifuge tubes. The samples were immediately returned to the freezer, maintaining the tissues frozen to avoid an additional freeze–thaw. Brain samples were processed as described below for homogenization and genetic screening for NNV.

### 2.2. Viral Cell Culture Assays

In 2020, for general virus screening not related to NNV, pooled spleen and kidney were processed for virus isolation at the Animal Health Diagnostic Laboratory, Department of Agriculture, Ewing, New Jersey. Tissues were homogenized in Hanks’ balanced salt solution (HBSS) at a volume of 9:1 (HBSS:tissue), followed by centrifugation. The supernatant was diluted 1:1 with antibiotic media and incubated for at least 2 h at 4 °C. Three cell lines were used for general virus screening, including Bluegill fry-2 (BF-2), Epithelioma papulosum cyprini (EPC), and Chinook salmon embryo-214 (CHSE-214). The cells were grown in 24-well plates and within 24 h of confluence were seeded in duplicate with 100 µL of the centrifuged supernatant and incubated at 20 °C. Cells were monitored every 3 days for 2 weeks, followed by blind passaging of the samples onto freshly grown cells for a further 2-week monitoring period. If no cytopathic effects (CPE) were noted in the cells within the 4-week period, then they were considered negative.

For samples that were positive for NNV by molecular methods (described below), the original tissue homogenate or parallel eye/brain tissue stored at −80 °C was used to attempt virus isolation specific to NNV. In 2020 and 2021, the frozen tissue samples were submitted to the National Veterinary Services Laboratory (NVSL), United States Department of Agriculture, Animal and Plant Health Inspection Service (Ames, Iowa). Tissue homogenates from each fish were individually inoculated onto striped snakehead-1 cell line (SSN-1) and a clone of the striped snakehead cell line (E-11), cell lines known to be permissible to NNV isolation [43], and incubated at 20 °C. A minimum of two blind passages were made and cells were monitored for CPE for a 28-day incubation period. In 2022, samples were processed at the Western Fisheries Research Center (Seattle, Washington). Previously frozen supernatant from processed tissue homogenates from 15 samples that showed the lowest cycle threshold (CT) values in rRT-PCR, ranging from 17.5 to 24.8, were selected for virus isolation by cell culture assays. Each sample was tested individually, without pooling. These homogenates were centrifuged at 12,000× *g* for 5 min and held on ice. The supernatant was collected and diluted 1:10 in L-15 Leibovitz media (Cytiva, Logan, UT, USA), followed by two additional serial dilutions (1:100 and 1:1000) in L-15. A volume of 200 µL of the three dilutions of each sample were inoculated onto freshly seeded SSN-1 cells pretreated with 7% Polyethylene glycol (PEG; Sigma-Aldrich, St. Louis, MO, USA) in L-15. Following inoculation, plates were subject to an adsorption step comprising centrifugation of cell culture plates (600 g) at 20 °C for 45 min. Following the adsorption spin, 1 mL of L-15 was added to each well. This was duplicated, such that one set of sample plates was incubated at 20 °C and a second set of plates incubated at 25 °C. Cells were monitored regularly for CPE for 14 days, followed by a blind passage onto fresh SSN-1 cells and monitoring for an additional 14 days. If no CPE was detected within the 28-day incubation period, then the samples were considered negative in cell culture.

### 2.3. Molecular Screening and Confirmation of Nervous Necrosis Virus

Upon thawing brain tissue samples, 900 µL of Gibco^©^ minimum essential medium (Gibco, Grand Island, NY, USA) and a 5 mm bead were added to each sample tube before homogenizing using a TissueLyser (Qiagen, Germantown, MD, USA) for 2 min at 20 hz. The homogenate was left on ice for 5 min and centrifuged at 3000× *g* for 5 min at 6 °C. The supernatant was removed to a clean 2 mL microcentrifuge tube. For RNA extraction, 50 µL of each sample was transferred to a 96-well plate and extracted using the KingFisher MagMax-96 Viral RNA Isolation Kit (Thermo Fisher Scientific, AM1836) run on the KingFisher Flex RNA extraction robot (Thermo Fisher Scientific), according to manufacturer’s directions. A positive and negative extraction control was run along with each plate. Positive control material included BFNNV amplified in the SSN-1 cell line, provided from an outside laboratory (Kennebec River Biosciences, Richmond, ME, USA). For NNV screening, a rRT-PCR assay [44] was used with slight modifications. Specifically, cDNA synthesis was conducted using a high-capacity cDNA reverse transcription kit (Thermo Fisher Scientific, Carlsbad, CA, USA, #4368814) according to manufacturer’s instructions and maintained at −20 °C until further use. For rRT-PCR, the QuantiNova Probe PCR Kit (Qiagen #208252) was used with primers known to amplify the four established NNV species [44], RNA2 FOR: 5′-CAA CTG ACA RCG AHC ACA C-3′ and RNA2 REV: 5′-CCC ACC AYT TGG CVA C-3′ at a concentration of 0.4 µM and the RNA2 PROBE: 5′ 6-FAM TY CAR GCR ACT CGT GGT GCV G-BHQ1-3′ at a concentration of 0.2 µM. Plates were run on an Applied Biosystems 7500 or an ABI 7500 Fast real-time PCR system (Thermo Fisher Scientific) with a positive amplification control and negative template control. Cycling conditions were either 50 °C for 30 min, 95 °C for 15 min, followed by 40 cycles of 94 °C for 15 s and 60 °C for 60 s, or a fast mode that included 95 °C for 2 min, followed by 40 cycles of 95 °C for 5 s and 60 °C for 5 s. Samples crossing the automatically designated threshold were identified as presumptively positive.

An independent molecular assay, an endpoint reverse transcription PCR (RT-PCR) assay targeting a conserved region of the RNA2 gene using VNNV1 and VNNV2 primers [45] was used with slight modifications (detailed below) to confirm presumptive positive findings. Samples were run on a Veriti thermocycler (Applied Biosystems). A Platinum Taq DNA polymerase master mix kit (Thermo Fisher Scientific, Carlsbad, CA, USA) was used where each reaction contained 1× PCR buffer, 0.2 mM dNTP mixture, 1.5 mM MgCl_2_, 3 µL of template cDNA, and 0.4 µM of each primer, with molecular grade water added up to a 50 µL reaction volume. Cycle conditions were 94 °C for 2 min, followed by 40 cycles of 94 °C for 15 s, 50 °C for 30 s, and 68 °C for 1 min, followed by final extension at 68 °C for 5 min and holding at 4 °C. PCR products were visualized via gel electrophoresis on 1.2% agarose E-gels (Thermo Fisher Scientific, Carlsbad, CA, USA) containing SYBR Safe DNA gel stain alongside an E-gel 1KB Plus Ladder to estimate amplicon size and a GeneRuler Low Range Ladder DNA Ladder (Thermo Fisher Scientific) to roughly estimate DNA quantity. PCR products that contained amplicons around 605 bp in size were selected for Sanger sequencing. In situations when no amplicons were detected, then the RT-PCR product was subject to nested RT-PCR using the primers VNNV3 and VNNV4 to amplify a 255 bp product according to a previously published protocol [45]. When the expected amplicon size occurred in either the first or nested round of RT-PCR, then the PCR product was processed for genetic sequencing, as detailed below. Genetic sequences were confirmed as NNV by comparing the sequences to those deposited in the National Library of Medicine, National Center for Biotechnology Information (NCBI), using the nucleotide basic local alignment search tool (BLASTn, https://blast.ncbi.nlm.nih.gov). Samples that were positive by rRT-PCR and confirmed with the independent endpoint RT-PCR assay combined with genetic sequencing were considered positive for NNV.

### 2.4. Genetic Sequencing

Various primer combinations (Table 1) were used to PCR amplify and sequence the RNA1 and RNA2 genomes of select samples. When necessary, new primers were designed using the Primer3 web-based software (Primer3web, version 4.1.0) [46], based on available genetic sequences from our study. For RT-PCR amplification, the master mix and primer concentrations described above were used. Cycling conditions described above or those matching the respective reference for the primers listed in Table 1 were used. When appropriately sized amplicons were detected by gel electrophoresis, the RT-PCR product was enzymatically purified using ExoSAP-IT (Thermo Fisher Scientific) according to the manufacturer’s directions. Sequencing reactions were prepared by diluting the purified DNA to approximately 2 ng/µL with molecular grade water, followed by the addition of 5 µL of the sequencing primer at a 5 µM concentration to make a total volume of 15 µL. The primers used for RT-PCR amplification were also used for sequencing. Sequencing was performed in both directions by Azenta (South Plainfield, NJ, USA) using ABI BigDye version 3.1 (Applied Biosystems) and run on an ABI 3730xl DNA analyzer (Applied Biosystems). Sequence quality was examined using Chromas Lite (Technelysium, Version 2.6.6) and sequence alignment was conducted using BioEdit (Version 7.2.5) to assemble sequences for the RNA1 and RNA2 segments.

To amplify and sequence the 5′ segment of the RNA2 in the new genotype from 2022, a representative sample (Sample ID: 22-137) was used to perform 5′RACE (Thermo Fisher Scientific) to obtain the genomic terminal sequences, as previously described [47]. The 5′RACE Kit protocol was followed for synthesis, purification, and for TdT tailing of the cDNA according to manufacturer’s directions and as previously reported [47].

**Table 1:** Primers used for the amplification and sequencing of the RNA1 and RNA2 segments of nervous necrosis virus. Unshaded were used for red-spotted grouper nervous necrosis virus, black shading was used only for the unique genotype in 2022, and gray shading was used for both genotypes. Nested PCR is shown by a first round PCR * followed by a nested PCR **. Intended amplicon size (Amp size) in base pairs.

Primer Name	Sequence	Target and Amp Size	Reference
RNA1_283-5′F	TAA CAT CAC CTT CTT GCT	RNA1 874	[48]
RNA1_856-874R	GGT GCT CAC CCA TCT TGA		
RNA1_684-705F	GAA CTA CAA CCA GGA TAC CAT G	RNA1 684	[48]
RNA1_1346-1367R	GAC TCA CTT GGA AAT ACA		
JRNV1_F1	TCA CTT ACG CAA GGT TAC CG	RNA1 1122	[49]
JRNV1_R1	GAC CGG CGA ACA GTA TCT GAC		
JRNV1_F1	TCA CTT ACG CAA GGT TAC CG	RNA1 1973	[49]
RNA1_1955-1973R	TGA CAG CAG GTG CTT GG		[48]
JRNV1_F2	AGT CTG GGY YTG GAR GGC	RNA1 1032	[49]
JRNV1_R2	GAC GAA AGC RTT DGC AAT GC		
VNNV5	GTT GAG GAT TAT CGC CAA CG	RNA1 953	[50]
VNNV6	ACC GGC GAA CAG TAT CTG AC		
FOR521	ACG TGG ACA TGC ATG AGT TG	RNA1 630	[51]
VNNV6	ACC GGC GAA CAG TAT CTG AC		[50,51]
RNA1_1248-1267F	CTT GCK CGT CAT TAC CAA GC	RNA1 839	This study
RNA1_2068-2087R	GCG ACC AGC AAG GTA TGA GA		
JRNV1_F3	TCC AAG CAC CWG CTG T	RNA1 1099	[49]
JRNV1_R3	GGG GTG GGA GCR GGC A		
BF Pol 1698-1715F	GTC CAG CTA CAC CTA CGC	RNA1 785	[48]
BF Pol 2465-2482R	AGT CTG CGT ATT GGA CCA		
RNA2_283-5′F	TAA TCC ATC ACC GCT TTG	RNA2 593	[48]
RNA2_578-597R	GCT GCC AAC ACA CAG GA		
RNA2_8-21F	TCA YCG CTT TGC MAT CAC AA	RNA2 421	This study
RNA2_410-429R	CGT TGT CAG TTG GAT CAG GC		
VNNV1 *	ACA CTG GAG TTT GAA ATT CA	RNA2 605	[45]
VNNV2 *	GTC TTG TTG AAG TTG TCC CA		
VNNV3 **	ATT GTG CCC CGC AAA CAC	RNA2 255	[45]
VNNV4 **	GAC ACG TTG ACC ACA TCA GT		
RNA2_818-837F	CAT TGA CTA CAA CCT TGG AG	RNA2 409	[48]
RNA2_1206-1227R	CAA TGG TAC CAA CAA TAG		

### 2.5. Phylogenetic Analysis of Nervous Necrosis Virus Sequences

Partial RNA1 and RNA2 consensus sequences were aligned and compared to reference nucleotide sequences retrieved from GenBank using the MEGA 7.0 package [52]. Reference sequences were selected according to the BLAST results obtained for each gene of each sample. Representative strains of each genotype were also included. For both RNA1 and RNA2 nucleic and amino acid alignments, phylogenetic relationships among strains were inferred using the maximum likelihood (ML) method available in the IQ-Tree software v1.6.9 [53]. The best fitting model of nucleotide (nt) and amino acid (AA) substitution was determined with ModelFinder [54]. One thousand bootstrap replicates were performed to assess the robustness of individual nodes of the phylogeny, and only values ≥70% were considered significant. Phylogenetic trees were visualized with the FigTree v1.4 software (http://tree.bio.ed.ac.uk/software/figtree/, accessed on 31 May 2025).

Phylogenetic trees based on partial nucleic sequence alignments were also developed for both genetic segments using the neighbor-joining (NJ) method with 1000 bootstrap re-samplings using the MEGA 7.0 package [52]. Pairwise similarities were also determined.

## 3. Results

### 3.1. Virology and Nervous Necrosis Virus Findings in 2020

Viral cell culture assays from spleen and kidney pools for general virus screening were negative for virus isolation in all three cell lines and incubation temperatures (Table 2). For NNV screening by rRT-PCR, all YOY black sea bass were negative. From adult fish, NNV was detected from one fish (Sample ID: 20-045) out of 258 fish screened (Table 2). This fish was collected from the Sea Girt Reef and had a total length of 220 mm and weight of 148.6 g. The detection was very low, having a CT of 35. Confirmation and genetic sequencing of this sample yielded a 1338 bp sequence of the RNA1 segment and 1226 bp sequence of the RNA2 segment, both deposited in GenBank (Table 3). A BLASTn search indicated that both segments have the closest identities (approximately 95–98% similarity; searched in July 2025) with various RGNNV sequences. An attempt to isolate this virus from previously frozen homogenates in the E-11 cell line failed to induce CPE.

### 3.2. Nervous Necrosis Virus Findings in 2021

A total of six presumptive NNV detections resulted from a total of 303 fish screened using rRT-PCR. Five fish, three male, and two females were confirmed positive with endpoint RT-PCR and sequencing (Table 2). Samples 21-252, 21-261, and 21-266 were confirmed positive with nested PCR [45] and partial sequences of the RNA2 gene (200–240 bp) confirmed their identity within RGNNV (Table 3). Fish 21-223 with a CT value of 38.9 failed to produce a reliable sequence, thus was not confirmed positive. Three of the detections came from the Sea Girt Reef and the other two from Little Egg Reef. The average total length and weight of fish testing positive for the virus was 263 ± 39 mm and 225 ± 77 g, respectively.

Partial RNA1 and RNA2 sequences were generated from these samples and deposited to GenBank (Table 3). Sequences spanning a large portion (93%) of the RNA1 sequence (2896 bp) and a majority (87%) of the RNA2 gene (1243 bp) were obtained for sample 21-182, while a large portion of only the RNA1 gene (95%) was obtained for sample 21-058 (2930 bp). BLASTn analysis of the RNA1 sequence for sample 21-058 showed the closest identity to RGNNV from red grouper *Epinephelus morio* in Taiwan with 97.47% identity (accession# FJ809938; BLAST search in July 2025) and around 96–97% identity with other RGNNV sequences in NCBI. Comparison of the RNA1 segments between samples 21-058 and 21-182 showed 97.76% similarity, with a total of 65 nucleotide substitutions between them, indicating considerable sequence variation in samples from the same fish population at the same point in time. BLASTn analysis of the RNA2 segment of 21-182 demonstrated a 97.1% match to an RGNNV isolate from China (accession# EF558369; BLAST search in July 2025). Comparison of the RNA2 sequences between samples 20-045 and 21-182 showed a 98.61% identity with 17 nucleotide differences.

Previously frozen tissue homogenates from all five positive samples processed for virus isolation failed to induce CPE in SSN-1 and E-ll cell lines. Molecular detection of the virus from the first passage in the cell line was confirmed in sample 21-58 using endpoint RT-PCR [45], though this was likely not from virus amplification in cell culture, but rather detection of the virus carried over in the cell culture inoculum. The genetic sequence matched that of the previous sequence generated for that sample.

### 3.3. Nervous Necrosis Virus Findings in 2022

**Figure 2:**
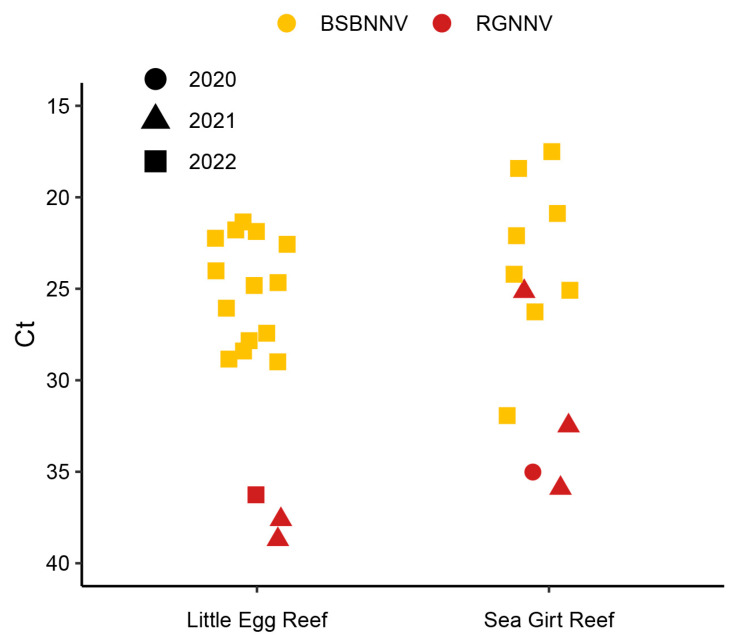
2020–2022 confirmed nervous necrosis virus reverse transcription real-time PCR (rRT-PCR) detections with cycle threshold (CT) values from black sea bass *Centropristis striata* collected from two reef sites. Note the reverse *Y*-axis scale showing cycle threshold values in rRT-PCR. Red-spotted grouper nervous necrosis virus (RGNNV); black sea bass nervous necrosis virus (BSBNNV).

A total of 82 presumptive positive detections of NNV occurred using rRT-PCR from a total of 304 fish collected in July. Of the 82 detections, 28 samples had a CT value under 30, 18 samples were between 30 and 35, and 36 samples had a CT value over 35 (Figure 2). The two lowest CT values were 17.5 and 18.4. A total of 11 out of those 28 samples with CT under 30 originated from the Sea Girt Reef, while 17 were from Little Egg Reef. This included 16 female and 12 male fish with a mean total length (TL) of 240 ± 38 mm and weight of 204 ± 96 g. Only a subset of samples was selected to confirm with endpoint RT-PCR and sequencing, including all 28 samples that had a CT under 30 and 2 additional samples. Of the 30 samples, a total of 23 were confirmed positive with endpoint RT-PCR and genetic sequencing. Representative sequences were deposited in GenBank (Table 3).

Genetic sequencing of the RNA1 yielded five samples (22-028, 22-045, 22-053, 22-191, and 22-273) with continuous sequences covering much of the RNA1 (about 2390 bp: about 77% complete). Sequences for 22-045, 22-053, and 22-273 were identical, whereas two nucleotide substitutions occurred in the other sequences. BLASTn comparison in NCBI indicates the closest identity of this RNA1 segment is with an isolate from Atlantic cod from Norway (Accession# EF577395; BLAST search performed in July 2025) with 91.24% identity and BFNNV from barfin flounder *Verasper moseri* from Japan (Accession# NC013458; BLAST search performed in July 2025) with 90.9% identity. The remaining 13 RNA1 sequences (Table 3) were partial or discontinuous sequences, ranging between 757 and 1950 bp in length. Alignment and comparison of the sequences indicated that all the partial RNA1 sequences had high identity to each other with rare single nucleotide substitutions or single nucleotide polymorphisms. Alignment of 12 partial sequences spanning 1022 nucleotides of the RNA1 correspond to nucleotide positions 80–1102 when aligned with the full RNA1 reference BFNNV sequence (accession# EU236146). Comparison of the sequences showed two single nucleotide polymorphisms at position 798 in three sequences and position 1005 in two sequences. Both nucleotide polymorphisms were a C-T substitution. Additionally, single nucleotide substitutions occurred in three sequences at positions 531, 723, and 912, which comprised a C-T, T-C, and A-G substitution, respectively.

Genetic sequencing of the RNA2 gene yielded partial sequences around 577 bp in length from 18 fish samples with the exception of 2 samples that only yielded sequences 250 bp in length. Two distinct sequence types were detected: a predominant sequence type (type I) that occurred in 17 samples and an infrequent sequence type II that occurred in 1 sample (22-228). The type I sequences were nearly identical to each other with rare single nucleotide substitutions detected. Fifteen of the partial RNA2 type I sequences (575 bp) were aligned along with the complete RNA2 gene from BFNNV (accession # EU236147). The partial sequences aligned with nucleotide positions 365 to 940 in this reference RNA2 sequence. This comparison showed five sequences that had a single nucleotide substitution, which included either an A-G, G-T, C-T, G-A, or T-C substitution. The other 10 sequences were identical. GenBank accession numbers are summarized in Table 3. 5′RACE techniques provided the sequence on the 5′ end of a single representative sample of the type I RNA2 (sample 22-137, accession # PV877396), which provided the most complete sequence of the RNA2 from this set of sequences (955 bp). BLASTn comparison of this sequence to other sequences published in NCBI showed closest identity (88.6%) to two submissions of TPNNV (accession # NC013461 and D38637; search performed in July 2025).

The type II RNA2 sequence from sample 22-228 (Table 3) was 546 bp in length. BLASTn analysis in NCBI indicates that 22-228 shares closest identity with various sequences including a viral isolate from Japanese flounder *Paralichthys olivaceus* (D38527), SJNNV (AY600956), viral isolate from European sea bass *Dicentrarchus labrax* (AF175510), and RGNNV (accession NC008041), all with around 95–96% identity. When this sequence was compared with the two RGNNV sequences from 2020 and 2021, sequence divergence was about 2.6%.

All viral cell culture assays on previously frozen tissue homogenates failed to induce CPE in the SSN-1 cell lines incubated at either 20 or 25 °C.

### 3.4. Phylogenetic Analysis of Nervous Necrosis Virus Sequences

**Figure 3:**
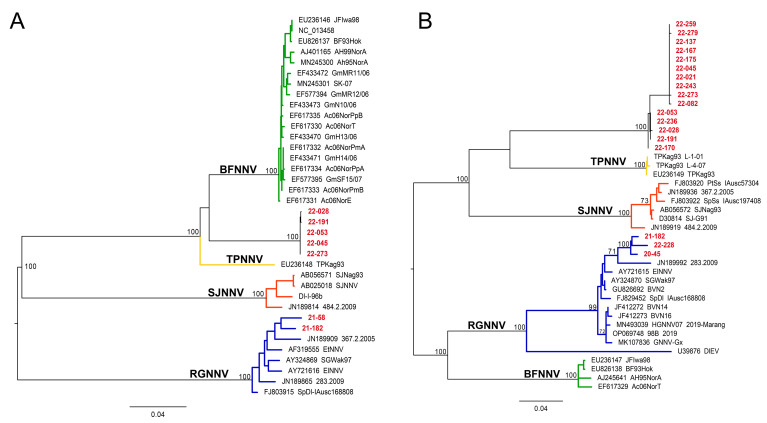
Maximum likelihood (ML) phylogenetic tree based on nucleotide sequences of the RNA1 (**A**) and RNA2 (**B**). Betanodaviruses collected from black sea bass *Centropristis striata* reported in this work are in red. The numbers at branch points represent bootstrap values expressed as percentages (only values ≥ 70 are reported). The genotype subdivision according to [7] is shown at the main branches. Scale bar represents the number of substitutions per site. Barfin flounder nervous necrosis virus (BFNNV) in green; tiger puffer nervous necrosis virus (TPNNV) in yellow; striped jack nervous necrosis virus (SJNNV) in orange; red-spotted grouper nervous necrosis virus (RGNNV) in blue.

Maximum likelihood phylogenetic analysis based on a selection of the longest nucleotide sequences for both RNA1 and RNA2 clearly grouped black sea bass viruses in two distinct viral clusters (Figure 3). The first cluster fell within the RGNNV genotype and included viral sequences obtained in 2020 (20-045—RNA1 and RNA2) in 2021 (21-058 and 21-182—RNA1 only) and 2022 (22-228—RNA1 and RNA2). The second cluster included only samples detected in 2022, which formed a new, homogeneous, and strongly supported group of sequences clustering separately from all previously described genotypes. This group is most closely related to BFNNV and TPNNV according to RNA1 and TPNNV and SJNNV according to RNA2 (Figure 3).

Indeed, when compared to TPNNV, RNA1 nucleotide identity ranged from 90.96% to 91.29% and amino acid (AA) identity ranged between 95.50 and 95.96%, while nucleotide identity to BFNNV ranged from 90.81% to 91.40% and AA identity ranged between 95.56 and 95.95%.

**Figure 4:**
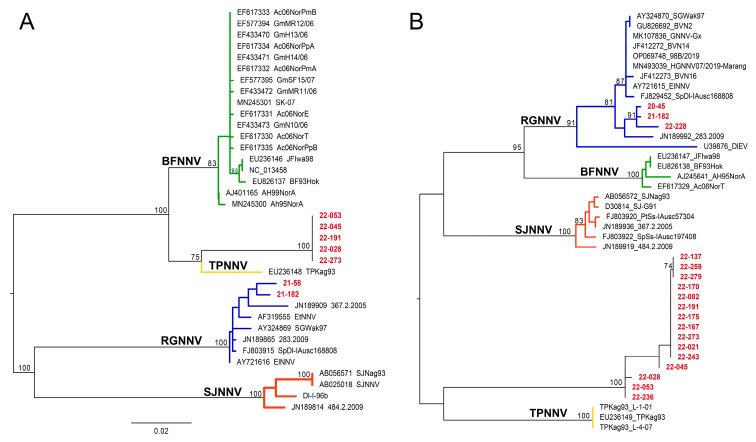
Maximum likelihood (ML) phylogenetic tree based on amino acid sequences of the RNA1 (**A**) and RNA2 (**B**). Betanodaviruses collected from black sea bass *Centropristis striata* reported in this work are in red. The numbers at branch points represent bootstrap values expressed as percentages (only values ≥ 70 are reported). The genotype subdivision according to [7] is shown at the main branches. Scale bar represents the number of substitutions per site. Barfin flounder nervous necrosis virus (BFNNV) in green; tiger puffer nervous necrosis virus (TPNNV) in yellow; striped jack nervous necrosis virus (SJNNV) in orange; red-spotted grouper nervous necrosis virus (RGNNV) in blue.

RNA2 nucleotide identity to TPNNV ranged from 82.74% to 83.43% and AA identity ranged between 83.33 and 83.51%. Nucleotide identity to SJNNV ranged from 81.03% to 81.54% and AA identity was 81.93–82.44%. Nucleotide identity to BFNNV was 77.55–78.07% and AA identity was 78.12–79.12%. The obtained findings were corroborated by the ML amino acid phylogenetic analysis on the same samples (Figure 4) and by the NJ phylogeny performed on a larger dataset (Supplementary Figure S1).

## 4. Discussion

These findings mark the first molecular detection of RGNNV within the North American Atlantic coast. This finding in black sea bass is consistent with previous detections of RGNNV, which are often found in groupers within the family Serranidae [22,24,31,49]. To our knowledge, the only other previous report of RGNNV in the United States was off the California coast associated with mortality in farmed white sea bass [27]. A notable finding herein was that the RGNNV sequences were unique from each other, having diverged between 1.4% and 2.6% within partial RNA2 sequences. This pattern probably does not indicate a single virus strain that has recently been transmitted or one that is circulating within the population, but rather could suggest individual fish carrying unique virus strains that likely originated from separate transmission events. The nucleotide substitution rate between samples is similar to that described for RGNNV from Europe [56] and Asia [57]. Though this is the first detection of RGNNV in the U.S. mid-Atlantic, the sequence diversity within these individual detections does suggest that it has likely been present long before this detection. Although no histology was available in this study to look for disease signs, the low prevalence of detection in the population, ranging from 0.66 to 3.3%, combined with the high CT values in rRT-PCR suggest very low virus levels in fish, as expected in “carrier” host stages that are not associated with clinical signs. The low prevalence and lack of sequence similarity also indicates that this virus was not transmitted actively among the population of black sea bass during the time of sampling. Further work to collect additional RGNNV sequence types in this region would be needed to learn more about RGNNV diversity to better confirm nucleotide substitution rates and to understand if there is regional clustering of sequence types as shown in other regions [56,57].

An unexpected finding in 2022 was a unique genotype that was not detected in the preceding two years. Indeed, phylogenetic analysis for both genetic segments, based on nucleotide and amino acid alignments, highlighted the presence of a new and strongly supported cluster, tentatively called black sea bass nervous necrosis virus (BSBNNV). This genetic uniqueness was also confirmed from the estimated pairwise nucleotide and amino acid distances showing clear divergence of this cluster from all other genotypes, while showing a relative closeness to the BFNNV and TPNNV genotypes. According to ICTV, in order to claim a new genotype of NNV, the nucleotide sequence of the newly detected genomic RNAs should be compared with those of other nodaviruses and show encoding capsid proteins that differ at >15% of nucleotides and >12% of amino acid positions [1]. In our case, the RNA2 of BSBNNV showed identities ranging from 77 to 83% for the nt sequence and from 78 to 83% for the AA sequence with the other known betanodaviruses—strongly supporting the discovery of a new viral genotype. Therefore, in addition to the turbot nodavirus (TNV) [17] and the SKNNV in shellfish [58], the herein described BSBNNV genotype from black sea bass could be considered in the next revision of the genus *Betanodavirus* taxonomy.

The unique BSBNNV showed a distinct detection pattern suggesting active infection and transmission of the virus in the black sea bass population. This is supported by the high prevalence in the population and detections by rRT-PCR with low CT values that were more indicative of heavy viral infections (two lowest CT values were 17.5 and 18.4). Although the rRT-PCR assay used was not a quantitative method, the relative differences in the CT values observed in 2022 compared to those found in RGNNV infected fish during the earlier two years is notable and likely related to virus concentration in the tissue samples. Although standard curves associating virus concentration and CT value were not established herein, this same rRT-PCR assay was previously validated and a standard curve showed a linear relationship of CT and serial dilutions of in vitro transcribed RNA [44]. In this study, when brain tissue from fish showing clinical signs of VNN were tested using this assay then CT values ranged from 9.9 to 18.6 [44]. In addition to the relatively low CT values observed in 2022, when viral sequences from individual fish were compared, they were highly uniform indicating a single virus strain being transmitted within the population. This sequence uniformity is consistent with other reported NNV outbreaks within hatcheries when sequences are identical in samples collected over a similar point in time [57]. This indicates that the 2022 sampling had overlapped with a natural epizootic with this novel genotype. An important question that remains unanswered is related to the virulence of BSBNNV in wild black sea bass. Viral nervous necrosis has been reported to cause disease in adult groupers in the wild, as seen by loss of equilibrium and lethargy, fin erosion, skin ulcerations around the head, swim bladder hyperinflation, and corneal opacity [30,31]. Samples for histology were unavailable to evaluate for neurologic lesions related to VNN and behavioral clinical signs would have easily been missed during sampling with the trapping method used. Further, it is not uncommon to observe some ulcerations and fin erosions from the traps, thus it may have been difficult to discern sampling injuries from viral specific lesions. The origin of this virus genotype is unknown, though it is notable to document its high prevalence and relatively high levels in the adult black sea bass population. Further, whereas RGNNV was detected at low levels in all three years, this novel genotype was only detected in 2022, which poses the question if this was a more recently introduced virus into this population. These questions may be further addressed with continued monitoring to learn more about the prevalence and sequence diversity of this genotype, along with the collection of parallel samples for histology and fresh tissues to further attempt virus isolation and evaluate the virulence of this genotype.

The presumptive and confirmatory detection methods for NNV in this study were molecular based, using independent rRT-PCR, endpoint RT-PCR, and genetic sequencing to confirm the presence of viral genetic sequences directly from fish brain tissue. These methods do not confirm the presence of replicating intact virus, which requires isolation of the virus in cell culture. Herein, previously frozen tissue homogenates from rRT-PCR positive fish and confirmed positive samples for RGNNV failed to isolate the virus in the SSN-1 and E-11 cell lines. This may be explained by multiple factors, including the expected low levels of virus based on rRT-PCR CT values (25.1–38.7) combined with the use of frozen tissue homogenates that were exposed to at least two freeze–thaw cycles. The novel genotype detected in 2022 also failed to be isolated in SSN-1 cells. Considering that the CT values in rRT-PCR suggested heavy virus infection, other factors need to be considered for isolating this virus. The novel genotype here is closely related to BFNNV based on sequence identity and phylogenetic analysis. It has previously been reported that BFNNV from Atlantic halibut *Hippoglossus hippoglossus* failed to replicate in the SSN-1 cell line even when fresh tissues from clinically affected fish were used. This was evidenced by no CPE and a decrease in virus quantity as estimated by rRT-PCR CT values during cell culture incubation, which increased from 20.9 at inoculation to 34.8 by the end of the incubation period [59]. Further, another study on NNV from turbot *Scophthalmus maximus* (TNV) failed to isolate the virus from clinically affected fish within the SSN-1 cell line [17]. Though the SSN-1 cell line and associated clones (E-11) are considered permissive to the various genotypes of NNV [43], it is clear that some virus strains are not effectively isolated in this cell line. Considering that these are the first detections of NNV in this species and in this geographic range, isolating a replicating virus can help further confirm risks associated with this virus. Based on the results herein, further work to identify a suitable cell line to isolate these NNV genotypes may aid in performing additional work to understand characteristics and virulence of this virus. Incubation temperature is an important consideration for in vitro isolation and cultivation of *Betanodavirus* spp. [9,10,11]. Considering that BSBNNV is closely related to BFNNV and TPNNV, which both replicate at relatively lower temperatures [43], 15–20 °C may be a good starting point for cultivating this virus.

As thermal constraints limit fish species and population ranges, these similar constraints influence NNV replication and prevalence, as seen by temperature preferences for the different genotypes [43]. Temperature influence has been shown to be regulated by the RNA1 segment encoding an RNA-dependent RNA polymerase [10], with optimal replication temperatures for BFNNV being 15–20 °C, RGNNV at 25–30 °C, and SJNNV and TPNNV in the middle of this range [43]. The black sea bass collected from this study are part of the mid-Atlantic population, which range from Cape Hatteras, North Carolina, north to the Gulf of Maine. The mid-Atlantic population historically reached only as far north as Massachusetts, though because of warming sea temperatures their range has recently expanded to the Gulf of Maine [60,61]. This recent range expansion for black sea bass into the Gulf of Maine is significant, as the Gulf of Maine is where BFNNV has been reported in coldwater species, including winter flounder, haddock, and Atlantic cod [13,14,26]. It is possible that fish range expansions could lead to new fish species interactions, which may influence virus transmission and evolution. These fish species interactions and life history are important to consider, particularly for NNV, as this is an RNA virus with a wide host range that may adapt through evolution and reassortment of RNA1 and RNA2 gene segments, as has been shown between RGNNV and SJNNV genotypes [8,62]. Considering that the novel BSBNNV described herein is closely related to BFNNV, it would be curious to see if the ranges of these genotypes overlap in the Gulf of Maine and if that could influence the interaction or evolution of these genotypes. Additionally on the southern end of the range, Cape Hatteras, North Carolina, is considered a major biogeographical boundary due to a sharp thermal change in this region [37]. This boundary divides the mid-Atlantic black sea bass populations from the south-Atlantic population, which are genetically distinct and range from Cape Hatteras, North Carolina, south to Florida, though some limited mixing between the populations may occur in North Carolina [37,63]. It would be interesting to know if similar NNV genotypes exist in the south-Atlantic population, or if the thermal barriers and limited interactions prevent exchange of viruses between these populations. Considering that RGNNV replicates well in warm conditions (25–30 °C), it would not be surprising if that genotype occurs in the south-Atlantic population. Monitoring that population and other fish species from these regions will shed light on the molecular epidemiology of NNV within the U.S. Atlantic coast.

As black sea bass are a prime candidate species for marine aquaculture [34,64], the potential of NNV infection in this species suggests that biosecurity may be important to avoid future disease issues. As this is a species relatively new to aquaculture, practices involve the use of wild-captured broodstock held in recirculation systems [34,65,66]. Vertical transmission has been suggested for NNV and this can lead to heavy mortality of larval progeny [67,68], suggesting that these disease risks may exist when utilizing wild collected black sea bass broodstock. As with other marine fish larvae, black sea bass larvae are sensitive to environmental factors in early life stages and mortality is not uncommon [69]. Poor early survival believed to be related to environmental factors may obscure mortality related to NNV if not properly investigated. With the findings herein, NNV could be a consideration in the list of rule-outs related to larval or juvenile mortality of black sea bass. As NNV has been shown to impact marine aquaculture in diverse fish species in many regions, the findings here suggest that implementing biosecurity plans to prevent NNV in marine aquaculture practices in this region could help prevent disease issues. Further, continued monitoring of wild fish and developing methods to isolate these viruses in cell culture could help to better understand NNV virulence in commercially important fish species.

## 5. Conclusions

Two genotypes of NNV have been detected using molecular methods for the first time in black sea bass collected off the coast of New Jersey, USA. These findings suggest an expanded range for red-spotted grouper nervous necrosis virus (RGNNV) to include the U.S. Atlantic coast. Further, a novel genotype tentatively named black sea bass nervous necrosis virus (BSBNNV) has been genetically characterized from this region. As NNV is known to cause major disease issues in marine aquaculture worldwide, these findings highlight the need for additional research to better understand virulence and epidemiology of NNV in this region.

## Figures and Tables

**Table 2:** Black sea bass *Centropristis striata* collected from the coast of New Jersey for general viral screening by virus isolation in cell culture (VI) or two-step reverse transcription real-time PCR (rRT-PCR) for nervous necrosis virus. Total length (TL) in mm and fish weight in g ± standard deviation; young-of-the-year (YOY). The number of confirmed detections/total fish are indicated.

Sampling Dates	Life Stage	Fish TL	Fish Weight	Method	Results
2020—August–October	Adult	246 ± 46	204 ± 113	VI cell culture	0/146
2020—March–August	YOY	62 ± 11	NA	rRT-PCR	0/116
2020—August	Adult	246 ± 46	203 ± 112	rRT-PCR	1/132
2020—October–November	Adult	239 ± 50	203 ± 120	rRT-PCR	0/126
2021—April	Adult	259 ± 25	220 ± 52	rRT-PCR	0/32
2021—July	Adult	244 ± 38	193 ± 96	rRT-PCR	2/180
2021—October	Adult	253 ± 38	225 ± 87	rRT-PCR	3/91
2022—July	Adult	262 ± 44	258 ± 150	rRT-PCR	23/304

**Table 3:** Samples confirmed positive for nervous necrosis virus by sequencing of the RNA1 and/or RNA2 genes. Associated GenBank accession numbers are provided. Short* are short or discontinuous sequence lengths not submitted to GenBank, but data are available in a U.S. Geological Survey data release [55]; (-) indicates no sequence was generated.

Year	Strain ID	RNA1	RNA2	Year	Strain ID	RNA1	RNA2
2020	20-045	PV877385	PV877386	2022	22-167	PV993951	PV993960
2021	21-058	PV877388	-		22-170	PV993952	PV993961
	21-182	PV877387	PV877389		22-175	PV993953	PV993962
	21-252	Short*	Short*		22-178	Short*	-
	21-261	-	Short*		22-191	PV877392	PV993963
	21-266	-	Short*		22-203	Short*	-
2022	22-021	PV993948	PV993956		22-213	Short*	-
	22-028	PV877390	PV877394		22-228	-	PV877397
	22-045	PV877391	PV877395		22-236	PV993954	PV993964
	22-053	PV993949	PV993957		22-243	Short*	PV993965
	22-062	PV993950	Short*		22-259	-	PV993966
	22-082	-	PV993958		22-273	PV993955	PV993967
	22-086	Short*	-		22-279	-	PV993968
	22-137	Short*	PV877396		22-298	PV877393	-
	22-163	-	Short*				

## Data Availability

Genetic sequences have been deposited to the National Center for Biotechnology Information (NCBI) under accession numbers PV877385–PV877398 and PV993948–PV993968. Metadata and raw data from this study are available through the U.S. Geological Survey ScienceBase at the following: Lovy, J. and Batts, W.N., 2025, Surveillance for nervous necrosis virus in black sea bass from the U.S. Atlantic coast: U.S. Geological Survey data release [55], https://doi.org/10.5066/P17MAMLV.

## Supplementary Materials

The following supporting information can be downloaded at: https://www.mdpi.com/article/10.3390/v17091234/s1, Figure S1: Neighbour-joining (NJ) phylogenetic trees.

## Abbreviations

The following abbreviations are used in this manuscript:

NNV	Nervous necrosis virus
VNN	Viral nervous necrosis
BFNNV	Barfin flounder nervous necrosis virus
RGNNV	Red-spotted grouper nervous necrosis virus
SJNNV	Striped jack nervous necrosis virus
TPNNV	Tiger puffer nervous necrosis virus
BSBNNV	Black sea bass nervous necrosis virus
rRT-PCR	Real-time reverse transcription polymerase chain reaction
RT-PCR	Endpoint/conventional reverse transcription polymerase chain reaction
ICTV	International Committee on Taxonomy of Viruses
CPE	Cytopathic effects
BF-2	Bluegill fry-2 cells
EPC	Epithelioma papulosum cyprini cells
CHSE-214	Chinook salmon embryo-214 cells
SSN-1	Striped snakehead-1 cells
E-11	Clone of striped snakehead cells
CT	Cycle threshold
PEG	Polyethylene glycol
HBSS	Hanks’ balanced salt solution
MEM	Minimum essential medium
nt	Nucleotide
AA	Amino acid
NCBI	National Center for Biotechnology Information
TL	Total length
YOY	Young of the year
BLAS	Nucleotide basic local alignment search tool
