# Supplementary material for: Detection and Genetic Characterization of Red-Spotted Grouper Nervous Necrosis Virus and a Novel Genotype of Nervous Necrosis Virus in Black Sea Bass from the U.S. Atlantic Coast
Source: Viruses. 2025 Sep 10;17(9):1234. doi: 10.3390/v17091234 (PMC12474048; doi:10.3390/v17091234)
Supplement: Supplementary file 1 [file viruses-17-01234-s001.zip › viruses-3849600-supplementary.pdf]

viruses

Supplemental Figures

Detection and Genetic Characterization of Red-Spotted Grouper Nervous Necrosis Virus and a Novel Genotype of Nervous Necrosis Virus in Black Sea Bass from the U.S. Atlantic Coast

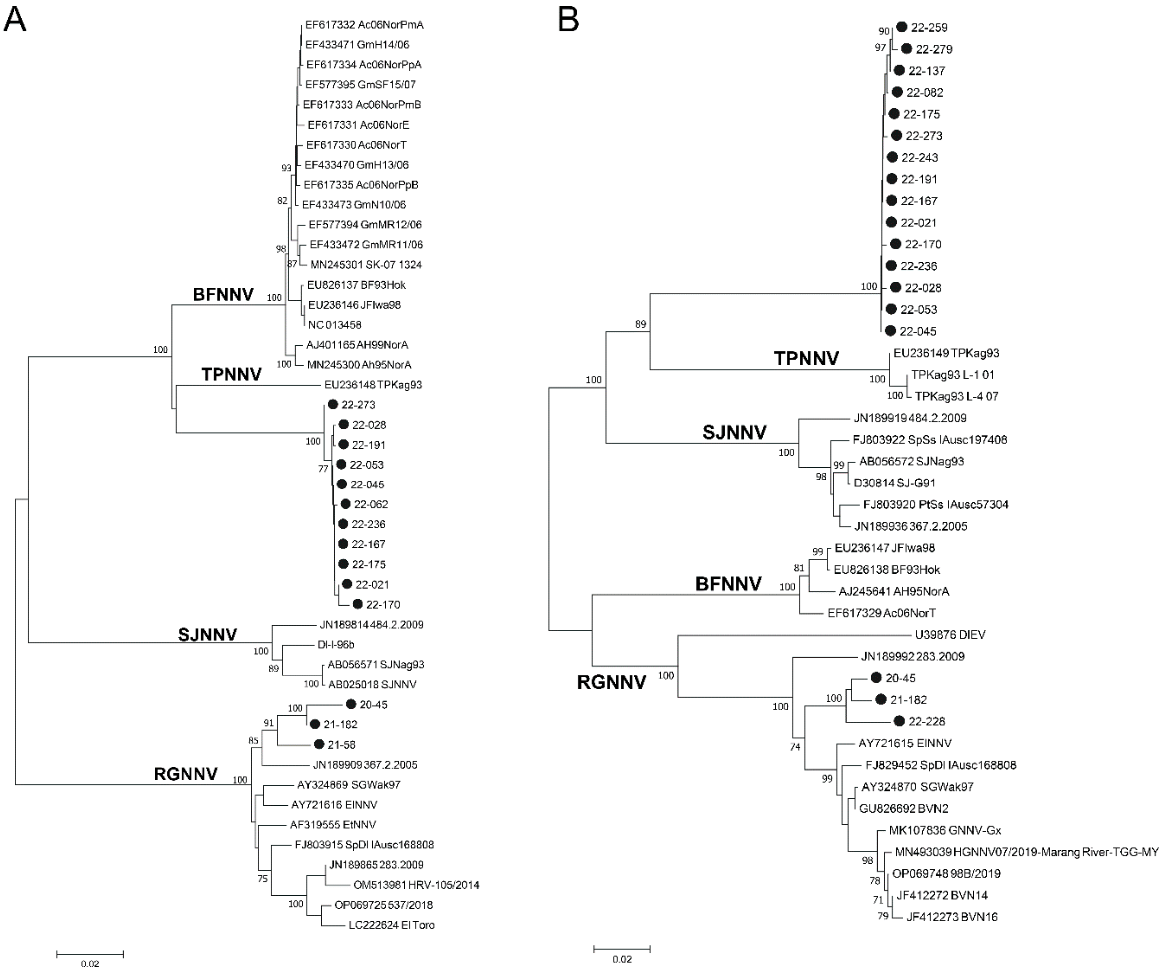

Supplemental Figure S1. Neighbour-joining (NJ) phylogenetic trees. (A) partial RNA1-based phylogeny. (B) Partial RNA2-based phylogeny. Betanodaviruses collected between 2020 and 2022 from black sea bass *Centropristis striata* reported in this work are labelled with a black circle. The numbers at branch points represent bootstrap values expressed as percentages (only values ≥ 70 are reported). The genotype subdivision according to [1] is shown at the main branches. Scale bar represents nucleotide substitutions per site. Barfin flounder nervous necrosis virus (BFNNV); tiger puffer nervous necrosis virus (TPNNV); striped jack nervous necrosis virus (SJNNV); red-spotted grouper nervous necrosis virus (RGNNV).

References

1. Nishizawa, T.; Furuhashi, M.; Nagai, T.; Nakai, T.; Muroga, K. Genomic classification of fish nodaviruses by molecular phylogenetic analysis of the coat protein gene. *Applied and environmental microbiology* **1997**, *63*, 1633-1636.
